# Safety, efficiency, and treatment satisfaction in children with primary immunodeficiency receiving subcutaneous immunoglobulin treatment

**DOI:** 10.14744/nci.2020.16870

**Published:** 2022-07-20

**Authors:** Sevgi Bilgic Eltan, Ozlem Keskin, Mehmet Fatih Deveci

**Affiliations:** 1 Division of Pediatric Allergy and Immunology, Department of Pediatrics, Gaziantep University Faculty of Medicine, Gaziantep, Turkiye; 2 Department of Pediatrics, Gaziantep University Faculty of Medicine, Gaziantep, Turkiye

**Keywords:** Child, primary immunodeficiency, subcutaneous immunoglobulin therapy

## Abstract

**OBJECTIVE:**

Patients with Inborn Errors of Immunity, also known as Primary Immunodeficiency (PID), are prone to recurrent bacterial infections and these patients often require lifelong IgG replacement therapy. The aim of this presentation is to evaluate the efficacy, safety, and patient satisfaction in PID patients receiving subcutaneous immunoglobulin (SCIG) treatment and to share our expe-riences.

**METHODS:**

Twenty-one patients who were followed up with the diagnosis of PID by our Pediatric Allergy and Immunology Clinic and received regular intravenous immunoglobulin therapy (IVIG) befo-re starting SCIG treatment were included in the study.

**RESULTS:**

A total of 21 patients were included in the study. 10 of the patients (47.6%) were female, 11 (52.4%) were male, and the mean age was 8.8±4.42 years. Five of the patients were Syrian patients living in the refugee camp. Threshold IgG levels of the patients were evaluated every 3 months. IgG levels were significantly higher than baseline IVIG levels at weeks 3, 6, and 12 of SCIG treatment, respectively. There was no significant difference between 3^rd^, 6^th^ and 12^th^ months of SCIG treatment. A statistically significant decrease was observed in the frequency of infections in patients who received SCIG treatment (p=0.003). During SCIG treatment, the total infection rate was 4.1/person/year. According to the TSQM-9 satisfaction questionnaire, the annual hospitalization rate was 0.9/patient/year for IVIG and 0.4/patient/year for SCIG (p>0.005), and 61.9% of patients were moderately satisfied, 14.2%. 19% were very satisfied and 4.7% were not satisfied with the treatment. When the satisfaction criteria were evaluated, it was observed that the patients mostly (71%) were satisfied with the absence of vascular access prob-lems and the comfort of self-application at home.

**CONCLUSION:**

SCIG therapy causes high serum IgG levels and a reduced frequency of infections and can be a safe, effective, and well-tolerated treatment alternative in patients with PID with high patient satisfaction.

## Highlight key points

- Our clinical experience suggests that SCIG therapy is a safe, effective, and valid alterna-tive, well-tolerated in the treatment of PID patients.
- The most important advantages for patients are the absence of vascular access problems, self-administration at home and short infusion time.
- Compliance of treatment and efficacy should be monitored with close clinical follow-up.

Children with primary immunodeficiency (PID) are prone to recurrent bacterial infections, and these patients generally require lifelong IgG replacement therapy [1, 2] as a result. Intravenous immunoglobulin (IVIG) therapy has been the standard care since the 1980s’ [3]. It has recently been understood that long-term IVIG administration has distinct disadvantages: [4] a large change in serum IgG concentrations due to monthly infusion of IVIG and the need for repeated venous entry have complicated this therapy for some patients [5, 6].

Because of the smaller doses administered at shorter intervals, subcutaneous immunoglobulin (SCIG) therapy is typically associated with less systemic reaction and higher serum IgG levels than IVIG [2, 7]. An additional benefit of SCIG is self-management at home, which generally has a positive effect on patients’ independence, vitality, and general health [8–10].

Increased tolerability of subcutaneous preparations formulated for intravenous (IV) use may be related to high purity, low potential for aggregation, and low viscosity. For convenience, it may be an additional benefit to continue on the same preparation mode that is effective and well-tolerated during IV administration for patients with PID wishing to switch to subcutaneous (SC) treatment [11].

This study intends to evaluate the treatment that has been used in Turkey over the last few years in terms of efficacy, safety, and patient satisfaction.

## MATERIALS AND METHODS

Twenty-one patients were followed up by our Pediatric Allergy and Immunology Clinic following a diagnosis of PID and underwent regular IVIG treatment before switching to SCIG treatment. These individuals were included in the study.

This study was carried out with a 10% IVIG preparation. IVIG product is a preparation of purified IgG antibodies containing solution for 10 g/100 mL IV infusion/SC use. At least 98% of the total protein is gamma globulin. The product is free of sugar or sodium and is suitable for use as an iso-osmolar to human plasma and stabilized with glycine.

### Study Population

Pediatric patients with a PID diagnosis according to the World Health Organization criteria, who had received regular IgG therapy (IV) at a dose of 400–1,000 mg/kg/4 weeks for at least 1 year, were included in the study.

### Study Design

The study consisted of three phases: patients who underwent SC treatment at a weekly dose equivalent to 137% of the IV dose had their IgG levels (with the turbidimetric method, COBAS INTEGRA 400 plus device), infection frequency, systemic and local side effect profile evaluated at 3, 6, and 12 months of treatment. A twelve-question patient questionnaire and questionnaire on drug treatment satisfaction (TSQM-9) were given to the patients on the 12^th^ month of the SC treatment.

TSQM is a questionnaire with 14 items consisting of four scales that are psychometrically reliable and valid [12]. The first of the four primary scales (questions 1–3) of the TSQM is the efficacy scale, the second (questions 4–8) is the side effects scale, the third (questions 9–11) is the compliance scale, and the fourth (questions 12–14) is the overall satisfaction scale. In our study, five items related to drug side effects were not included for TSQM-9. The TSQM-9 domain scores were calculated as recommended by the instrument authors, which is described in detail elsewhere [13, 14]. The TSQM-9 domain scores range from 0 to 100 with higher scores representing higher satisfaction on that domain.

Ethics committee approval with numbered 2016/22, dated 25.01.2016, was obtained from Gaziantep University Faculty of Medicine Ethics Committee for the study. Written informed consent was obtained from all patients before initiating any study procedure. Families of each patient provided written informed consent and all studies were conducted in accordance with the principles of the Declaration of Helsinki.

### Subcutaneous Administration

Initially, three SC infusions were initiated with parental education by an experienced nurse at the hospital. Patients who were convinced that they could do the administration on their own were switched to treatment at home. The infusion rate was increased from 5 mL/h/area to 15 mL/h/area and 20 mL/h/area for patients below and above 40 kg, respectively. Where this ratio was tolerated, subsequent infusions were made starting at 10 mL/h/area and a maximum of 20–30 mL/h/area. A infusion every 15–20 min was achieved for subjects weighing 40 kg and <40 kg, respectively. The average SCIG dose was obtained by calculating 137% of the case’s monthly doses. The maximum infusion rate was 1.5 mL/kg/h. The number of infusion sites ran-ged from one to two.

### Efficiency and Safety

The treatment efficacy was assessed by considering infection frequency and threshold IgG levels. Infections were classified as those requiring hospital admission and those with outpatient infections. Patients were informed about the infections and were questioned about the frequency of their infections occasionally, once a month, once every 2 months, and once every 3 months with a patient questionnaire.

The side effects were checked by calling the families 72 h after the infusion was completed. All post-infusion side effects were recorded by patients in a diary and recorded at regular intervals in the case report during scheduled clinical visits. Local side effects were rated as mild-moderate-severe.

### Statistical Analysis

The normality of the distribution of continuous variables was tested using the Shaphiro–Wilk test. The Kruskal–Wallis and all subset multiple comparison tests were used for comparison of three independent groups of variables with a non-normal distribution, the McNemar test was used to assess the relation between dependent categorical variables, and the Freidman test was applied to evaluate changes over time for non-normal numerical data. Statistical analysis was performed in SPSS (2020, IBM, New York/ABD) and a p<0.05 was accepted as statistically significant.

## RESULTS

### Profiles of Patients

A total of 21 patients were included in the study. 10 of the patients (47.6%) were female, 11 (52.4%) were male, and the mean age was 8.8 (±4.42). Of all cases, 33.5% (n=7) had combined immunodeficiency (5 of these cases were diagnosed with Bloom Syndrome during the study period), five had common variable immunodeficiency, 23.8% (n=5) had ataxia-telangiectasia, 9.5% (n=2) had hyper IgM syndrome, 4.7% (n=1) had Netherton Syndrome, and 4.7% (n=1) had Hyper IgE Syndrome. Five of the patients were Syrians living in a refugee camp.

### Efficiency

**Figure 1:**
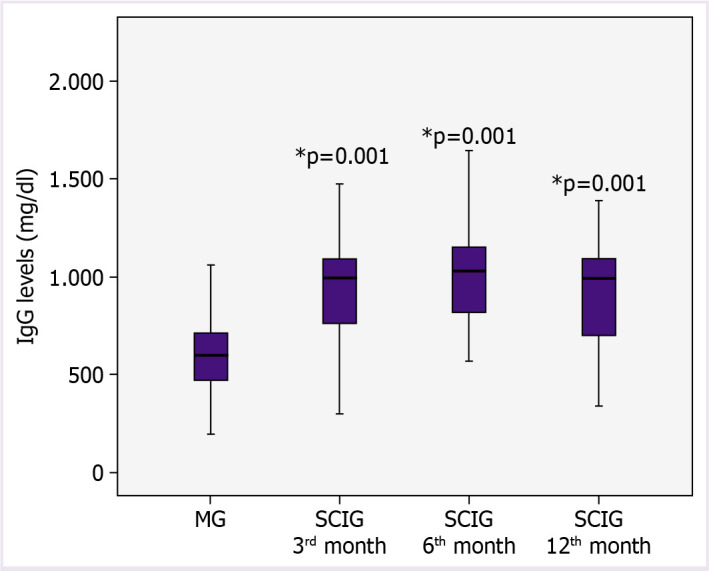
Comparison of IgG values over time (Freidman test, IVIG-3^rd^ month SCIG, IVIG-6^th^ month SCIG, IVIG-12^th^ month SCIG, p=0.001).

The median IgG levels at 3, 6, and 12 weeks of SCIG treatment were significantly higher than the baseline IVIG levels, respectively (955 mg/dl [IQR: 763–1090], 1029 mg/dl [IQR: 818–1150], 992 mg/dl [IQR: 702–1089], respectively; p=0.001). There was no significant difference in SCIG treatment between months 3, 6, and 12 when the immunoglobulin levels were compared (SCIG 3^rd^ month-SCIG 6^th^ month p=0.952, SCIG 3^rd^ month-SCIG 12^th^ month P: 0.591, SCIG 6^th^ month-SCIG 12^th^ month p=0.633) (Fig. 1).

When the frequency of infections requiring outpatient antibiotic treatment was examined, a statistically significant decrease in the frequency of infection was observed in patients receiving SCIG treatment (p=0.003). The total infection rate during SCIG was 4.1/year/person. The annual hospitalization rate was 0.9/patient/year for IVIG and 0.4/patient/year for SCIG (p>0.005) (Table 1).

**Table 1:** Safety and efficiency profile of IVIG and SCIG treatment

	IVIG (n=21) %	SCIG (n=21) %	p
Efficacy			
Hospitalization	0.9 patients/year	0.4 patients/year	**>0.05** **0.003**
Outpatient infection treatment (%)			
Occasionally	0	31.3	
Every 3 months	6.3	18.8	
Every 2 months	18.8	31.3	
Once a month	75	18.8	
Safety	n=21 (%)	n=21 (%)	
Local side effects	**0**	**95**	**0.001**
Mild		80.9	
Moderate		14.2	
Severe		0	
Swelling		76.2	
Rash		52.3	
Pain		33.3	
Systemic side effects	**80.9**	**0**	**0.001**
Headache	28.5		
Lethargy	14.3		
Fever	28.5		
Shivering	19		
Urticaria-pruritus	19		
Dyspnea	9.5		
Other	4.7		

IVIG: Intravenous immunoglobulin; SCIG: Subcutaneous immunoglobulin.

### Safety

The SCIG treatment was completed without dosage reduction or interruption due to 99.8% of all SC infusions being tolerable. No severe adverse effects with IVIG or SCIG were reported during the study. No local side effects were observed during the IVIG treatment (p=0.001). Patients switching to SCIG treatment had no systemic side effects, and 95% of the patients had local side effects (pain at the injection site, redness, swelling). There was a significant change in the observation of systemic side effects when the drug was changed (p=0.001) (Table 1).

### Treatment Satisfaction

According to the TSQM-9 satisfaction survey, 61.9% of the patients were satisfied with the treatment; a patient not responding to the treatment switched to the IVIG treatment at month 12. When the satisfaction criteria were evaluated, it was seen that the patients were most satisfied (71%) with the absence of vascular problems and the convenience of being able to self-administer at home (Table 2).

**Table 2:** Satisfaction percentages and patient satisfaction criteria in SCIG treatment

Satisfaction (%)	
I am satisfied with the treatment	61.9
I am somewhat satisfied	14.2
I am very satisfied	19
I am not satisfied	4.7
Satisfaction criteria (n=21) %	
I feel better	52
I am not having any side effects	57
I can self-administer at home	71
Infusion duration is shorter	66
I am not experiencing vascular problems	71
Financially advantageous	57

SCIG: Subcutaneous immunoglobulin.

According to the TSQM-9 survey, the overall scale score showed a satisfaction level of approximately 57%, while the highest satisfaction score was obtained from the efficacy subscale (65.2%). In addition, the scores from compliance and overall satisfaction scales are over 60 points (Table 3).

**Table 3:** Scores according to the TSQM-9 survey evaluation (n=20)

	Min.	Max.	Mean	SD
Efficiency	33	88	65.2	12.6
Compliance	22.2	88.8	61.6	15.9
Global Satisfaction	28.5	78.5	63.2	13.8
General Satisfaction	35.7	71.4	56.7	9.1

Min: Minimum; Max: Maximum; SD: Standard deviation.

## DISCUSSION

In our study, we aimed to determine the treatment efficacy, safety, and satisfaction of 21 patients who were switched from IVIG to SCIG. Clinical experience suggests that SCIG therapy is a safe, effective, and valid alternative, well-tolerated in the treatment of PID patients [1, 2, 7, 15].

SCIG is universally acknowledged by patients with a history of serious adverse events following IVIG infusion, such as renal disease and thromboembolic events, poor vascular access, and protein loss enteropathy. However, in some countries such as Sweden, the UK, and Germany, SCIG is the standard treatment for the majority of PID patients because of easy management, the lowered use of medical resources, reduction of direct and indirect costs, and improved quality of life.

Based on these advantages as demonstrated in many studies, it is reasonable to consider changing IgG therapy from IVIG to SCIG in willing patients who can self-administer at home.

In a review by Abolhassani et al. [16], it was clearly shown that acceptable IgG serum levels were reached with SCIG replacement therapy. Following IVIG infusion, the systemic distribution of IgG is much faster than it is after the application of SCIG [16]. With IVIG, an IgG peak is reached 15 min after completion of the infusion and the infused IgG is substantially reduced over the next 48 h. As a result, the IgG serum level continues to decline at first-rate kinetics with a half-life of 36±10.8 days [17], and IVIG infusion given every 21–28 days does not lead to constant Ig concentrations. Conversely, weekly SCIG administration leads to a peak level of IgG reached after 4–6 days of infusion due to slow release after injection and a half-life of 40.6±9.7 days [18]. In studies conducted in our country of Turkey, the coefficient of 1.37 was used for products with a concentration of 10% [19]. In our clinic, the total monthly dose was determined using the same coefficient. The mean IgG values during treatment with SCIG were significantly higher than those with IVIG treatment.

The primary aim of lifelong IgG replacement treatment is to prevent organ damage by decreasing the frequency and severity of infection [9]. Moreover, several studies have shown that higher IgG levels are more effective in reducing chronic lung disease, sinus infections, and pneumonia [20–22]. Although there was a statistically significant decrease in the frequency of infection in our study, there was no significant difference in the annual hospitalization rates.

SCIG products, which are very similar to IVIG formulations, appear to be very similar in efficacy of protection against severe bacterial infections in PID patients [2, 7, 15]. However, when controlled comparative clinical trials have not been conducted, it has been reported that various manufacturing differences may affect the tolerability of the patient. These include saccharose or other stabilizers, IgG concentration, sodium content, osmolality, and foreign substances (e.g., Xia factor and its effects on thromboembolic events) [23–25]. In our study, the majority (95.2%) completed the infusion without interruption for tolerability.

Numerous studies have shown that drug non-compliance increases in adolescents with chronic illness. An adolescent patient quit treatment with SCIG at month of 12 due to a compliance problem. For this reason, we think that better control and management following home treatment in this age group is required.

Local side effects per infusion were observed at 95%. These side effects were mild and moderate. No severe local reactions were observed after SC administration. Several studies [1, 7] have shown that as patients gain experience with SC infusions, the local side effect ratio decreases over the course of the study. In the study of Aydiner et al. [19] from Turkey, local side effects were rarely reported and showed a tendency to disappear with repeated doses.

Currently, only 20% of the subcutaneous IgG preparation is available for high doses in the US and Europe, but the development of SCIG preparations is likely to continue, and is expected to provide new generation immunoglobulin formulations with improved absorption, such as preinjection of recombinant human hyaluronidase prior to SCIG infusion [26]. This will allow the injection and absorption of IgG in larger volumes. Administered at similar doses and intervals to those obtained with IVIG infusion, it may be administered by way of the subcutaneous route, which allows the administration of SCIG. This way, it will be possible to assess the efficacy and safety of SCIG as an immunomodulator for patients with autoimmune and inflammatory diseases. Preliminary studies have shown promising results, suggesting that subcutaneous infusion of IgG is a safe and practical route of administration with the same efficacy as IVIG in autoimmune disorders [27].

Individuals with PID face a chronic illness that requires lifelong treatment. Patients with chronic illnesses have been described as target groups for structured training in relation to lifestyle and behavioral changes [28]. In most European countries, specialist centers currently have training programs for self-education, due to the fact that the parents and families of patients need to be equipped to administer the infusion to their children [29]. A very significant change over the past 25 years is that patients and their families have gone from being passive recipients of treatment and care, and instead transitioned into a healthcare team of experts in disease and treatment. Additionally, many studies have shown that self-infusion at home develops quality of life [30, 31].

It is very important to monitor self-infusions for home treatment and to help patients and their families by constantly supporting and improving their strategies in coping with problems, through establishing a system where patients and parents can easily contact the PID team [32]. We believe that a system of “nursing and health-care services” support for homecare will improve patient compliance and improve treatment success in our country.

Our study reveals the importance of self-care at home, due to reasons such as long distances between the patient’s city and a health-care facility (possibly in another city), low socioeconomic levels (especially the five Syrian patients living in refugee camps), and difficulties in getting to a health-care facility owing to neurological findings, such as ataxia-telangiectasia. Our study reveals the importance of home self-care for patients who have difficulties in reaching the hospital for various reasons (transportation from the surrounding cities and refugee camps, disease-related obstacles such as AT, low socioeconomic level). In addition, the reduced use of medical resources can directly and indirectly reduce costs and contribute to the country’s economic prospects. In our clinic, the most important factors affecting treatment satisfaction were not experiencing vascular problems, self-administration at home, and short infusion duration. In the study of Bienvenu et al. [33], satisfaction with the treatment setting for home-based SCIG was found to be at maximum, but no difference was found between IVIG and SCIG in patients with home-based IgRT. These results show that the feeling of independence at home significantly affects treatment satisfaction.

The fact that the number of patients is low and that costings have not been made are the major limitations of the study.

## Conclusion

SCIG treatment is an efficient, safe, and easy option which is suitable for individual administration. It can be performed at home in selected, willing patients without contraindications after the necessary training is given and informed consent is obtained. Treatment compliance and efficacy should be evaluated individually and dynamically by measuring serum stable IgG levels inter-mittently with close clinical monitoring. Self-infusion with SCIG ensures that the patient feels safe during infusion at home or at work and increases patient satisfaction.
